# Development of a Model for Preliminary Diagnosis of Human Granulocytic Anaplasmosis

**DOI:** 10.1089/vbz.2023.0032

**Published:** 2023-10-04

**Authors:** Iryna Ben, Olena Zubach, Alexander Zinchuk

**Affiliations:** Department of Infectious Diseases, Danylo Halytsky Lviv National Medical University, Lviv, Ukraine.

**Keywords:** human granulocytic anaplasmosis, Lyme disease, diagnosis, logistic regression, disease model

## Abstract

**Background:**

Human granulocytic anaplasmosis (HGA) is a vector-borne natural focal disease that is not officially registered in Ukraine. The first 13 cases of HGA in adults in Ukraine were identified in 2007. The purpose of our study was to develop a predictive model of HGA based on clinical and laboratory characteristics to develop a three-level standard case definition of HGA.

**Materials and Methods:**

Researchers examined 498 patients with suspected tick-borne infections and carried out a retrospective clinical and epidemiological analysis of 60 cases recruited from Lviv regional infectious disease hospitals. Logistic regression was used to create a model of the probability of the diagnosis of HGA depending on the presence of certain clinical and laboratory factors that, when examined, together may help to confirm a case of HGA. For logistic regression, eight clinical and laboratory factors were selected: history of tick bite, hyperthermia, signs of pharyngitis, changes in chest X-ray picture (enhancement of the pulmonary pattern and enlargement of the lung root boundaries), increased bilirubin (˃21 μmol/L), increased alanine aminotransferase (ALT ˃36 U/L), erythema migrans, and detected Lyme disease.

**Results:**

In the presence of all eight factors, the probability of HGA is 95.7%. When the five main signs are absent—signs of pharyngitis, changes in chest X-ray picture, increased bilirubin and ALT, and a history of tick bite—the probability of HGA in the patient dramatically decreases to 6.8%, meaning that HGA might be excluded.

**Conclusions:**

Based on the analysis of epidemiological, clinical, and laboratory signs, criteria for establishing a suspected, probable, and confirmed diagnosis of HGA have been developed to improve diagnosis.

## Introduction

Human granulocytic anaplasmosis (HGA) is an acute tick-borne infection caused by the bacterium *Anaplasma phagocytophilum* and transmitted by *Ixodes* ticks (Bakken and Dumler, 2015; Dumic et al., 2022; Stuen et al., 2013). Since 1997, fewer than 300 cases of HGA have been recorded in Europe (Matei et al., 2019), with *Ixodes ricinus* as the main disease reservoir, and *Ixodes persulcatus* reported as a significant vector in Eastern Europe (Asman et al., 2019; Azagi et al., 2020; Berzina et al., 2013; European Centre for Disease Prevention and Control, 2022a). *Ixodes persulcatus* is known to be present in Ukraine (Akimov and Nebogatkin, 2016; European Centre for Disease Prevention and Control, 2022b). In the United States, HGA is included in the list of diseases subject to required reporting (Centers for Disease Control and Prevention, 2020; Ismail et al., 2010).

According to the Centers for Disease Control and Prevention (CDC), between 2000 and 2019, the number of registered HGA cases increased from 348 to 5655 per year (Centers for Disease Control and Prevention, 2020). In Europe, the official numbers of HGA cases in humans are undercounted, because reporting is voluntary, although it is understood that there is a high prevalence in animals (Gandy et al., 2022).

In 2007, the Research Institute of Epidemiology and Hygiene of Danylo Halytsky Lviv National Medical University conducted a retrospective study of 92 sera samples from patients with a history of tick bites from different regions of Ukraine. Among them, 13 cases of HGA were identified in Zaporizhzhya (4), Volyn (3), Cherkasy (2), Dnipro (1), Lviv (1), Poltava (1) and Rivne (1) Oblasts (Ben and Biletska; 2008, Biletska and Ben, 2008), which became the first HGA cases in adults reported in Ukraine. HGA cases were also occasionally reported in Kharkiv Oblast in 2010 (Malyi et al., 2010).

Despite the long-term study of tick-borne diseases in Ukraine, HGA cases have not been routinely reported, and it is still one of the less-known diseases among health care practitioners (Klymnyuk et al., 2017; Morochkovskyi and Ben, 2015). This is unsurprising, given that HGA transient infection may occur in the absence of associated clinical signs; subsequently, cases may not always be detected. Coinfection with other tick-borne pathogens should be investigated for HGA, especially for cases where a rash has been present (European Centre for Disease Prevention and Control, 2022a).

According to our data (Ben and Biletska, 2015) obtained in Western Ukraine, up to 50% of HGA cases were associated with coinfection with Lyme disease (LD). In addition, there are difficulties in diagnosing HGA due to a lack of laboratory capabilities, a streamlined system, and sufficient availability of enzyme-linked immunosorbent assay (ELISA) and PCR test kits to diagnose HGA in Ukraine (Kylypko et al., 2009). Considering the above, as well as the lack of HGA awareness among physicians and patients (Klymnyuk et al., 2017; Morochkovskyi and Ben, 2015), a prediction model based on clinical presentation and a general laboratory examination will be useful for the diagnosis and selection of treatment. Another important task is to determine clinical and laboratory features of HGA and statistically justify the list of basic signs that need to be considered first for HGA diagnosis (Centers for Disease Control and Prevention, 2016).

Clinical prediction models based on logistic regression have been used to diagnose HGA in China (Durant and Peaper, 2016) and Lyme meningitis in children in Rhode Island (Garro et al., 2009). We used a similar approach to develop a model for the clinical prediction of HGA based on clinical and general laboratory characteristics without specific testing for the presence of the pathogen or antigens (Lotrič-Furlan et al., 2015). Our model became the basis for a recommended three-level case definition of HGA.

## Materials and Methods

### Ethics statement

The study protocol was approved by the Medical Ethics Committee of the Danylo Halytsky Lviv National Medical University, Ukraine (protocol No. 6/2018). Patients provided written consent for the study before enrollment.

### Study subjects

A retrospective clinical and epidemiological study was conducted in two Oblast (regional) centers in Western Ukraine, Lviv and Lutsk, for 2007 through 2015. We examined clinical records of patients with suspected tick-borne diseases, and who had received inpatient treatment in Lviv regional infectious disease hospitals.

We included patients >18 years of age with the following inclusion criteria, where a patient needed to exhibit at least one criterion from each of the following three categories:

1. Epidemiological criteria: history of tick bite or visiting areas endemic for naturally focal infections, for example, forests, steppe, and mountains (Lozynski et al., 2013).
2. Clinical criteria (one or more of the following):
  a. Fever or signs of intoxication: weakness, fatigue, headache, myalgia.
  b. Skin lesions: spotty pink rash rarely papule or pustule on the limbs, which later extended to the torso and face.
  c. Signs of liver damage: liver enlargement, icteric sclera, increased levels of liver enzymes (transaminases).
  d. Changes in blood count: leukopenia, left shift of leukocyte formula, thrombocytopenia.
3. Absence of viral hepatitis B and C markers by ELISA method.

The following information was collected about each patient: age, sex, place of residence, history of tick bite, clinical features, laboratory examination (blood count and biochemical blood tests), chest X-ray examination, results of laboratory examination for infections, and treatment.

### Diagnosis confirmation

The confirmation of HGA diagnosis was performed using ELISA and PCR in accordance with recommendations of the *Rickettsia*, *Coxiella*, *Anaplasma* (*Ehrlichia*), and *Bartonella* research group of the European Society of Clinical Microbiology and Infectious Diseases (Brouqui et al., 2004; Lotrič-Furlan et al., 2015).

In the first stage of our study, sera samples from all patients were tested in ELISA for the presence of antibodies (IgG) to *A. phagocytophilum* in diagnostic titers (>1:100) using commercial kits (Omnix Ltd., St. Petersburg, Russia). At the second stage, the diagnostic test kit Ampli-HGA (Omnix Ltd.) was used to detect *A. phagocytophilum* DNA in the positive samples.

The diagnosis of HGA was considered confirmed when the patient had antibodies (IgG) to *A. phagocytophilum* in diagnostic titers (>1:100) and confirmed availability of *A. phagocytophilum* DNA in blood.

In addition, a commercial ELISA test kit (Omnix Ltd.) was used to detect IgG to *Borrelia* to study potential HGA-LD coinfection. Of the 498 patients, we examined only 452 patients due to resource constraints.

### Model development and testing

Logistic regression was used to create a model that predicts the probability of laboratory-confirmed HGA based on the presence of certain clinical and laboratory diagnostic factors.

For logistic regression, eight HGA clinical and laboratory diagnostic factors were selected: (1) history of a tick bite; (2) increase in body temperature (with patients placed into two groups, that is those with *t* up to 37.9° C; and those with *t* > 38° C); (3) signs of pharyngitis; (4) changes in the chest X-ray image (enhancement of the pulmonary pattern, that is, greater lung opacity and enlargement of the lung root boundaries, that is, hilar enlargement); (5) increase in blood bilirubin and (6) increase in alanine aminotransferase (ALT) level; (7) the presence of erythema migrans (EM); and (8) confirmed LD.

To evaluate the influence of the model on the variance regarding the diagnosis of HGA, the values of the Nagelkerke's R-square were assessed. To assess the accuracy of the model, a classification table was used to compare the difference in the percentage of predicted and real values. To determine the validity of the model, we used the chi-squared and the derivative value *p* with the significance level *p* < 0.05. The reliability of this model (*p*) is 0.003, with the chi-squared 23.629 with the degree of freedom = 8. Nagelkerke's R-square is 0.332, that is, 33.2% of the variance can be predicted using this model. At the same time, a retrospective review of our database showed that the percentage of correctly predicted cases was 80.7% (Harrell, 2015; Hendriksen et al., 2013; Lang and Secic, 2006). All calculations were performed using PSPP v.1.0.1.

## Results

The initial database included 90 patients matching the inclusion criteria with a complete clinical and laboratory profile. HGA was laboratory-confirmed in 60 patients but unverified in the remaining 30 patients. Only 30 (50% ± 6.5%) confirmed HGA patients had *A. phagocytophilum* monoinfection and 30 (50% ± 6.5%) patients with coinfection HGA and LD. EM was present in 36.7% ± 6.2% of the patients with laboratory-confirmed coinfection HGA and LD. The main clinical features in the patients with HGA are described in Table 1.

**Table 1.** Frequency of Clinical Signs in 60 Patients with Confirmed Human Granulocytic Anaplasmosis

Clinical signs	Frequency	
	Abs. number	M ± m (%)
Systemic intoxication syndrome		
Fever	42	70.0 ± 5.9
Asthenia	22	36.7 ± 6.2
Headache	19	31.7 ± 6.0
Lack of appetite	8	13.3 ± 4.4
EM	22	36.7 ± 6.2
Musculoskeletal system impairment		
Arthralgia	14	23.3 ± 5.5
Myalgia	13	21.7 ± 5.4
Gastrointestinal system impairment		
Tongue covered with white fur	20	33.3 ± 6.1
Epigastric pain	4	6.7 ± 3.2
Nausea	3	5 ± 2.3
Vomiting	3	5 ± 2.3
Liver impairment		
Icteric sclera and skin	7	11.7 ± 4.1
Dark urine	4	6.7 ± 3.2
High bilirubin level	12	20.0 ± 5.2
High transaminase level	12	20.0 ± 5.2
Respiratory system impairment		
Signs of pharyngitis	12	20 ± 5.2
Cough	4	6.7 ± 3.2
Abnormalities on chest X-ray image	12	20 ± 5.2
Heart impairment		
Tachycardia	7	11.7 ± 4.1
Nervous system impairment		
Sleep disorder	4	6.7 ± 3.2
Meningeal signs	2	3.3 ± 1.5
Leukocytosis	22	36.7 ± 6.2
Leukopenia	24	40 ± 6.3

EM, erythema migrans.

The results of the modeling showed that in the presence of all of the eight HGA clinical and laboratory diagnostic factors described above, the probability of the laboratory confirmation of HGA was 95.7% and increases to 97.2% when the body temperature reaches >38°C (Table 2).

**Table 2.** Results of Regression Coefficients in Relation to the Probability of Diagnosis of Human Granulocytic Anaplasmosis by the Method of Logistic Regression

No.	Clinical and laboratory diagnostic indicators (factors)	Regression coefficient (*R*)	Probability of HGA diagnosis (%)
1	All eight factors	3.096	95.7
2	Eight factors at febrile body temperature (*t* > 38°C)	3.551	97.2
3	Seven factors without history of tick bite	−0.598	45.9
4	Seven factors without EM	6.148	99.8
5	Seven factors without increase in body temperature (*t* − *N*)	2.641	93.4
6	Seven factors without manifestations of pharyngitis	1.411	80.4
7	Seven factors without increased bilirubin levels (bilirubin levels − *N*)	1.861	86.5
8	Seven factors without increased ALT levels (ALT levels − *N*)	2.426	91.9
9	Seven factors without changes in chest X-ray image	4.664	99.1
10	Seven factors without confirmation of LD in the patient	1.516	82.0

ALT, alanine aminotransferase; HGA, human granulocytic anaplasmosis; LD, Lyme disease.

Exclusion of one or more of the factors in the presence of the others significantly changed the probability of diagnosing HGA. Thus, the highest probability of confirmed HGA (99.8%) was observed in the presence of seven factors and the absence of EM (Table 2). A very high probability of confirming HGA was also observed in the presence of six factors and the absence of laboratory-confirmed LD and the absence of EM (99.0%) (Table 3). In the presence of seven factors, including EM, and the absence of only laboratory confirmation of LD, the probability to confirm HGA was lower than that for all eight factors (82.0%) (Table 2).

**Table 3.** Logistic Regression: Results of Regression Coefficients Regarding the Probability of Diagnosis of Human Granulocytic Anaplasmosis in the Absence of the Diagnostic Factor—Laboratory-Confirmed Lyme Disease

No.	Clinical and laboratory diagnostic indicators (factors)	Regression coefficient (*R*)	Probability of HGA diagnosis (%)
1	Six factors without the history of tick bite	−2.178	10.2
2	Six factors without EM	4.568	99.0
3	Six factors without increase in body temperature (*t* − *N*)	1.061	75.3
4	Six factors without manifestations of pharyngitis	−0.169	45.9
5	Six factors without increased bilirubin levels (bilirubin levels − *N*)	0.281	57.0
6	Six factors without increased ALT levels (ALT levels − *N*)	0.846	70.0
7	Six factors without changes in chest X-ray image	3.084	95.6

The probability of confirmed HGA in the presence of seven factors and the absence of changes on the chest X-ray picture (99.1%) exceeded the probability in the presence of all eight factors (Table 2). A slightly lower probability was associated with the presence of six factors and no changes on the chest X-ray picture and in the absence of laboratory-confirmed LD (95.6%), exceeding the effect of laboratory-confirmed LD as an independent factor (Table 3).

**FIG. 1.**
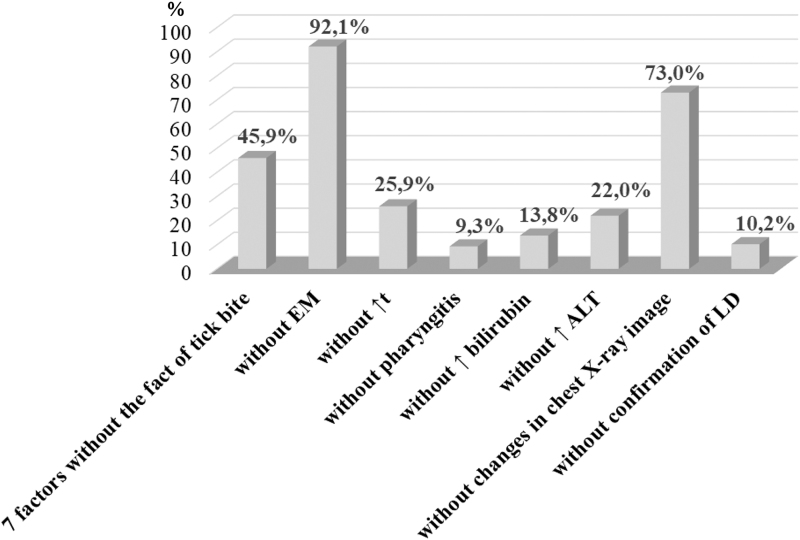
The probability of HGA diagnosis in a person with the absence of a single diagnostic factor without the history of tick bite. ALT, alanine aminotransferase; EM, erythema migrans; HGA, human granulocytic anaplasmosis; LD, Lyme disease.

The lowest probability of confirmed HGA in the presence of seven factors was 45.9% in cases when a patient denied or did not remember having had a tick bite (Fig. 1). It was even lower when a patient had normal body temperature or ALT level (25.9% and 22.0%, respectively), normal bilirubin level (13.8%), in the absence of confirmed LD (10.2%), and the lowest—in the absence of pharyngitis (9.3%) (Table 4). However, when the absence of history of tick bite was combined with already proven factors associated with a higher probability of confirmed HGA, namely, absence of migrating erythema and absence of changes in the chest X-ray picture, the probability of HGA confirmation increased up to 92.1% and 73.0%, respectively (Fig. 1).

**Table 4.** Logistic Regression: Results of Regression Coefficients Regarding the Probability of Diagnosis of Human Granulocytic Anaplasmosis in the Absence of the Diagnostic Factor—Tick Bite

No.	Clinical and laboratory diagnostic indicators (factors)	Regression coefficient (*R*)	Probability of HGA diagnosis (%)
1	Six factors without EM	2.454	92.1
2	Six factors without increase in body temperature (*t* − *N*)	−1.053	25.9
3	Six factors without manifestations of pharyngitis	−2.283	9.3
4	Six factors without increased bilirubin levels (bilirubin levels − *N*)	−1.833	13.8
5	Six factors without increased ALT levels (ALT levels − *N*)	12.268	22.0
6	Six factors without changes in chest X-ray image	0.98	72.95
7	Six factors without confirmation of LD in the patient	−2.178	10.2

**FIG. 2.**
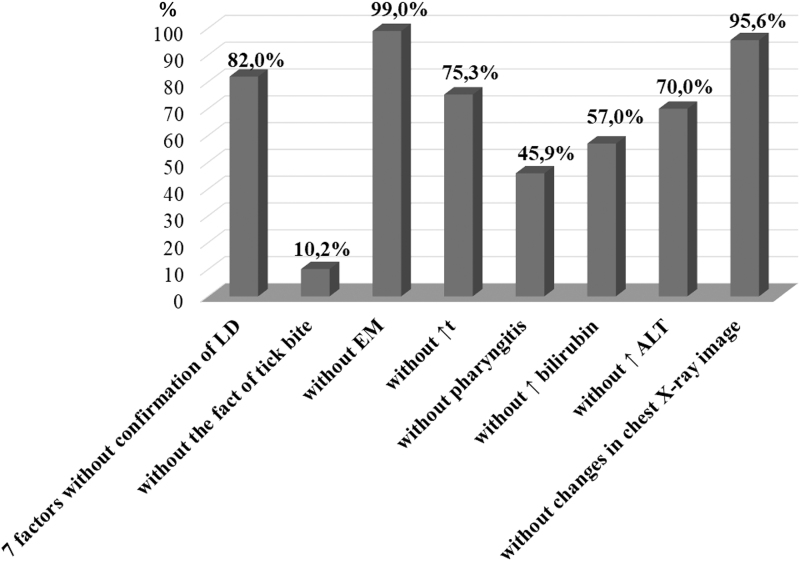
The probability of HGA diagnosis in a person with the absence of a single diagnostic factor without confirmation of LD in the patient.

According to our data (Ben and Biletska, 2015) obtained in Western Ukraine, up to 50% of HGA cases were associated with coinfection with LD. Therefore, the LD is also known to be an important factor. As mentioned above, with the combined effect of seven factors in the absence of laboratory-confirmed LD, the probability of confirming the diagnosis of HGA does not exceed 82.0%. Therefore, variants were calculated with the effect of six factors in the absence of laboratory-confirmed LD and one of the other factors (Fig. 2).

Based on the multifactor model, calculations of the importance of clinical and laboratory manifestations of HGA and combinations of them were made, and criteria for diagnosis were established. A three-level standard for defining the HGA case was proposed in accordance with established criteria:

1. Suspected HGA case (the probability of a diagnosis based on our calculations is <90%): intoxication syndrome (nonspecific symptoms)—subfebrile or febrile fever in the presence of one or more clinical signs: fatigue, general weakness, headache, lack of appetite, myalgia, arthralgia of large joints.
2. Probable case (the probability of diagnosis based on our calculations is above 90%): suspected case with specific clinical and laboratory signs (liver margin is 1–2 cm outside the costal arch, icteric sclera, increased liver enzymes [aminotransferases] level), and epidemiological criteria (tick bites or time spent in endemic areas, forest, etc.).
3. Confirmed case: laboratory confirmation from any one of the four criteria below must be added to the signs typical for a probable case (Centers for Disease Control and Prevention, 2021): (1) serological signs: fourfold increase of antibody titer (IgM, IgG) to *A. phagocytophilum* in paired blood serum in indirect immunofluorescence assay or ELISA (the first [acute] blood serum sample is collected in the first week of the disease, with the second [convalescent] sample) 2–4 weeks later; (2) *A. phagocytophilum* DNA detection in blood by PCR; (3) detecting the *A. phagocytophilum* antigen in the sample collected during biopsy/autopsy by available methods; or (4) the isolation of *A. phagocytophilum* from a clinical specimen on a cell culture.

## Discussion

Our article describes analysis of clinical signs and laboratory data of patients with laboratory-confirmed HGA in Western Ukraine. We determined the most typical signs of HGA based on the selected criteria. Using logistic regression, we developed a multifactorial model to determine the probability of HGA diagnosis. In the presence of all eight major factors (tick bite, presence of erythema, increased body temperature, signs of pharyngitis, increased levels of blood bilirubin and ALT, abnormalities found on chest X-ray image, and LD), the probability of HGA diagnosis was 95.7%.

Also, we have considered all possible variants of the prediction for the diagnosis of HGA in the absence of one of the factors, but the combination of the other seven of the above factors was retained. The highest probability to confirm the diagnosis of HGA is 99.8%, occurring in the presence of seven factors and the absence of erythema in the patient, exceeding the prediction of 95.7% under the influence of all eight factors, and once again confirming that EM serves as a pathognomonic sign of LD. It should be noted that, with the exclusion of each of the factors, the lowest probability to confirm the diagnosis of HGA, 45.9%, arose in the absence of a history of a tick bite.

One should consider that the fact of tick bite was confirmed by only 68.3% of patients with confirmed HGA. Other patients did not remember or did not notice the bite, and only one patient denied it. Tick bites can be located on the scalp or in areas that are hard to inspect and can be made by nymphs. Therefore, it is important to consider the presence in endemic areas. Most often, patients indicated tick bites during a walk, picking mushrooms and berries in the forest, or working in backyards or summer houses located near the forest. In the cities, tick bites happened in recreation areas, namely parks, urban forest belts, and school stadiums. The survey revealed that only one-third (12 patients) sought medical attention to remove ticks; the rest of the patients removed ticks themselves.

In view of these facts, it is important to simulate the absence of two factors, that is, the combined effect of six factors instead of eight. We calculated the probability of diagnosis of HGA in the absence of a factor—laboratory-confirmed LD and each factor in turn (Table 3). High probability of confirming the diagnosis of HGA (99.0%) occurred in the absence of both laboratory-confirmed LD and EM. The lack of laboratory-confirmed LD and the absence of a history of tick bite reduce the probability of confirming an HGA diagnosis to 10.2%. Therefore, we think that it is efficient to test for HGA in the patients who are positive for LD, taking into account the significant percentage of coinfection with HGA-LD (Ben and Biletska, 2015; Dvořáková Heroldová and Dvořáčková, 2014; Nieto and Foley, 2009), as well as patients who are negative for LD based on the results of the model.

Also, we calculated the probability of diagnosis of HGA in the absence of two factors—a history of tick bite and one of the other factors from our list. The maximum probability of HGA diagnosis is 92.1% when there is no EM and no data on the tick bite, and a minimum of 9.3% with the absence of tick bite in the recorded history and manifestations of pharyngitis. This emphasizes the importance of a careful collection of the patient's history.

If the patient has only EM, increase in body temperature, and laboratory-confirmed LD, but there are no characteristic manifestations of HGA (manifestations of pharyngitis, changes in chest X-ray image, changes in bilirubin and ALT with the tick bite in history), the prediction of HGA presence is 6.8%, and the probability of HGA diagnosis is minimal.

The diagnosis of known infectious diseases, and especially the detection of cases of new infections, requires the establishment of a diagnosis of the disease. Taking into account the experience of foreign colleagues (Bakken and Dumler, 2015; Dumic et al., 2022; Moniuszko-Malinowska et al., 2021; Pace and O`Reilly, 2020), calculations of the importance of clinical and laboratory manifestations of HGA and their combinations were made, and the criteria for diagnosis were established based on the developed multivariate model. A three-level standard for defining the HGA case was proposed in accordance with established criteria. This three-level diagnosis allows clinicians to establish a “suspected case” of the disease and establish a preliminary diagnosis to reduce the number of laboratory tests, prescribe treatment, and facilitate the recovery of the patient.

### Limitations

HGA is not officially monitored in Ukraine, and we do not have a full picture of morbidity and cannot calculate the model using official report data. We did not include demographic factors, as well as epidemiological data about visiting geographic locations with a high risk of tick bite (in case a patient did not mention or did not recall a history of a tick bite) in the model. In the future, we plan to evaluate the effectiveness of treatment depending on the timing, severity, and clinical form of the disease.

## Conclusions

The use of our model for prediction of the probability of HGA allowed the determination of the most important manifestations of HGA to improve the diagnosis of this tick-borne transmissible disease.
